# Disruption of Firmicutes and Actinobacteria abundance in tomato rhizosphere causes the incidence of bacterial wilt disease

**DOI:** 10.1038/s41396-020-00785-x

**Published:** 2020-10-07

**Authors:** Sang-Moo Lee, Hyun Gi Kong, Geun Cheol Song, Choong-Min Ryu

**Affiliations:** 1 Molecular Phytobacteriology Laboratory, Infectious Disease Research Center, KRIBB, Daejeon, 34141 South Korea; 2 Department of Biosystems and Bioengineering, KRIBB School of Biotechnology, University of Science and Technology, Daejeon, 34113 South Korea; 3 Present Address: Crop Protection Division, National Institute of Agricultural Sciences, Rural Development Administration, Wanju-gun, 54875 South Korea

**Keywords:** Plant sciences, Microbiome

## Abstract

Enrichment of protective microbiota in the rhizosphere facilitates disease suppression. However, how the disruption of protective rhizobacteria affects disease suppression is largely unknown. Here, we analyzed the rhizosphere microbial community of a healthy and diseased tomato plant grown <30-cm apart in a greenhouse at three different locations in South Korea. The abundance of Gram-positive Actinobacteria and Firmicutes phyla was lower in diseased rhizosphere soil (DRS) than in healthy rhizosphere soil (HRS) without changes in the causative *Ralstonia solanacearum* population. Artificial disruption of Gram-positive bacteria in HRS using 500-μg/mL vancomycin increased bacterial wilt occurrence in tomato. To identify HRS-specific and plant-protective Gram-positive bacteria species, *Brevibacterium frigoritolerans* HRS1, *Bacillus niacini* HRS2, *Solibacillus silvestris* HRS3, and *Bacillus luciferensis* HRS4 were selected from among 326 heat-stable culturable bacteria isolates. These four strains did not directly antagonize *R. solanacearum* but activated plant immunity. A synthetic community comprising these four strains displayed greater immune activation against *R. solanacearum* and extended plant protection by 4 more days in comparison with each individual strain. Overall, our results demonstrate for the first time that dysbiosis of the protective Gram-positive bacterial community in DRS promotes the incidence of disease.

## Introduction

Rhizosphere microbiota play an important role in plant fitness, development, and immunity [1–4]. The negative and positive effects of monocropping-induced soil microbial changes on plant health have been studied for a long time [5–8]. Monocropping alters the soil environment to facilitate disease progression in plants via a phenomenon known as negative plant–soil feedback [7]. On the other hand, after continuous and severe disease outbreak, monocropping also suppresses the progression of soil-borne diseases [5, 6]. Disease-suppressive soil was defined as soil with minimal disease incidence, despite the coexistence of virulent pathogens and susceptible plant hosts [5]. Disease-suppressive soil was mainly effective against soil-borne fungal pathogens such as *Rhizoctonia solani*, *Pythium ultimum*, *Gaeumannomyces graminis* var. *tritici*, *Plasmodiophora brassicae*, and *Fusarium oxysporum*, and a bacterial pathogen *Ralstonia solanacearum* [9–15]. In continuous monocropping systems, disease-suppressive soil can be induced by altering beneficial microbial communities in the rhizosphere [1, 16].

Rhizosphere microbes play a complex role in the establishment of disease-suppressive soils. Early studies on diseasesuppressive soils focused mainly on the direct mechanism of microbes against a target pathogen. The best example of an antagonistic microbe in disease-suppressive soil is the fluorescent pseudomonads, which directly inhibited the growth of *G. graminis* var. *tritici* by producing the antibiotic 2,4-diacetylphloroglucinol [6, 17]. On the other hand, the elicitation of induced systemic resistance (ISR) contributes indirectly to disease suppression [18–20]. ISR referred to the activation of immunity in the entire plant against a broad spectrum of pathogens by a rhizobacteria inoculated at a spatially distant site rather than at the site of pathogen infection [19, 20]. ISR is primarily regulated by jasmonic acid (JA) and ethylene (ET) signaling in *Arabidopsis* and tomato [21, 22]. Certain rhizobacteria can also trigger salicylic acid (SA)-dependent elicitation of ISR in plants [23, 24].

Most studies on disease-suppressive soils have focused on the effect of a selected microbial strain, rather than that of a microbial consortium, on the target pathogen [1, 6, 17, 25, 26]. Diverse beneficial rhizobacterial genera have been identified as disease-suppressing microbes including the genus of *Pseudomonas* [1, 17, 25], *Bacillus* [27, 28], *Paenibacillus* [29], and *Streptomyces* [14, 26]. Over the last decade, advances in next-generation sequencing approaches revealed that disease-suppressive soil is formed by the orchestrated action of a microbial complex rather than by a single microbial strain [1–4]. Recently, to mimic a natural disease-suppressive community, introduction of a synthetic community (SynCom), comprising multiple microbial strains, into germ-free or non-suppressive soil has been attempted [2, 30, 31]. However, most exogenous microbes lack the ability to survive in and colonize the rhizosphere or to protect the host plant under field conditions [32]. Thus, to design an artificial disease-suppressive SynCom, it is vital to understand and maintain homeostasis between the introduced SynCom and the pre-existing microbial community in the rhizosphere [32–34].

The eubiosis of host-associated microbial communities can potentially alter disease occurrence [35–39]. In animals, dysbiosis of gut microbiota, with respect to the composition, quantity, diversity, and metabolism of microbial populations, shows a strong correlation with inflammatory bowel disease, irritable bowel syndrome, neocritical disorders, and colorectal cancer [37–40]. Thus, microbial dysbiosis can lead to other pathological conditions [41]. In plants, network analyses reveal differences in the abundance of rhizosphere microbial communities between disease-suppressive and -conducive soils [26, 42, 43]. However, the effect of the disruption of specific protective bacteria on the rhizosphere is largely unknown. Dysbiosis of the phyllosphere microbiota was recently reported to cause disease on *Arabidopsis* leaves [44]. Reduction of Firmicute caused by an increase of the antagonistic Proteobacteria population was the main indication of dysbiosis.

Here, we examined the rhizosphere of two adjacent paired tomato plants with and without disease symptoms grown within a 30-cm distance in a greenhouse in three different geographic locations in South Korea. Both plants showed drastic differences in the incidence of bacterial wilt, despite the presence of a similar amount of the causal pathogen, *Ralstonia solanacearum*, in their rhizosphere. Analysis of the rhizosphere samples by 16S rRNA amplicon sequencing and cultivation-based approaches revealed a decline of Firmicutes and Actinobacteria in the diseased rhizosphere soil (DRS). Strikingly, the disruption of Firmicutes and Actinobacteria in the healthy rhizosphere soil (HRS) using the antibiotic vancomycin led to the DRS-mimicking phenomenon. Furthermore, a SynCom of HRS-specific strains activated JA signaling-dependent ISR against *R. solanacearum* in tomato. Based on our results, we suggest that dysbiosis of the protective microbial community in the soil disrupts disease suppression. Our results will help to broaden the agricultural applications of synthetic microbial communities as biological control agents against plant pathogens.

## Materials and methods

### Experimental setup and sampling

HRS and DRS samples were collected from three tomato plastic greenhouses, one each in Damyang (35°15′53.4″N and 126°55′11.8″E), Yongin (37°06′20.6″N and 127°08′14.3″E), and Gwangju (37°29′24.1″N and 127°18′22.6″E), in South Korea. Each greenhouse was 6.6 × 100 m in size and contained 950–1200 tomato plants. The temperature in each greenhouse was maintained at 30 ± 5 °C. Tomato plants in the selected DRS samples showed severe bacterial wilt symptoms including stem blight; wilting of petioles, main stem, branch tips, and leaves; and chlorosis and necrosis of foliage. Tomato plants in HRS samples did not show bacterial wilt symptoms and were adjacent to DRS samples (within a distance of 30 cm). The HRS and DRS were filtered through a 2-mm mesh to remove large soil particles and collected only rhizosphere soil without plant root tissue and debris. Then, the HRS and DRS were suspended in sterile distilled water for 30 min to remove tightly attached soil particles. The soil solution was centrifuged at 8000 rpm for 10 min, and the soil pellet containing the microbiome was stored at −80 °C until needed for microbial community analysis.

### Extraction of soil microbiota

Soil microbiome was extracted from HRS and DRS samples as described previously [45]. Briefly, HRS and DRS fractions (1 g/mL each) in 2.5-mM 2-(n-morpholino) ethanesulfonic acid (MES) monohydrate buffer were applied to the root system of 14-day-old tomato seedlings for 30 min using the root-dipping method. The HRS/DRS fraction-treated tomato plants were transplanted in sterilized soil and inoculated with *R. solanacearum*, the causal organism of bacterial wilt. At 10 days post inoculation (dpi), the severity of bacterial wilt was recorded on a 0–5 scale [46], with 0 indicating no symptoms, 1 indicating one partially wilted leaf, 2 indicating one to two wilted leaves, 3 indicating two to three wilted leaves, 4 indicating four or more wilted leaves, and 5 indicating the death of the entire plant.

### Disease suppression by the extracted soil microbiota

Seedlings of the bacterial wilt susceptible tomato variety, Juiken, were cultivated in sterile soil for 14 days after germination. Roots of tomato seedlings were treated with soil microbial fractions using the root-dipping method. On day 14, the treated roots were washed with distilled water to remove the attached soil particles and were immersed in 20-mL soil fraction for 30 min. Then, tomato seedlings were transplanted in sterilized soil and grown at 28 °C for 5 days. To inoculate tomato seedlings with the pathogen, *R. solanacearum* was grown in casamino acid-peptone-glucose (CPG) broth (1-g/L casamino acids, 10-g/L peptone, and 5-g/L glucose) at 30 °C for 24 h, and 10-mL pathogen suspension (OD_600_ = 1) was applied to the soil by drench application. All experiments were performed in triplicate, with 12 plants per treatment.

### Structure of the tomato rhizosphere microbiome

Microbial genomic DNA was extracted from HRS and DRS samples using the FastDNA Spin Kit (MP Biomedicals, Irvine, CA, USA) and quantified using Epoch Spectrometer (Biotek, VT, USA). PCR amplification was performed using primers targeting V3 and V4 regions of 16S rRNA genes. The first round of amplification was carried out using primers 341F and 805R (Table S1) under the following conditions: denaturation at 95 °C for 30 s, annealing at 55 °C for 30 s, and extension at 72 °C for 5 min. Secondary amplification was performed to attach the Illumina NexTera barcodes using primers i5-F and i7-R (Table S1) under the same amplification conditions as described above; however, the number of amplification cycles was set to eight. The PCR products were separated by electrophoresis on 1% agarose gel and visualized using a Gel-Doc system (Bio-Rad, Hercules, CA, USA). Then, the PCR products were purified using the CleanPCR Kit (CleanNA, Waddinxveen, the Netherlands), and equal concentrations of the purified products were pooled together. Nontarget short fragments were removed using the CleanPCR Kit, and the quality and size of PCR products were assessed using the DNA 7500 chip on Bioanalyzer 2100 (Agilent, Palo Alto, CA, USA). Pooled amplicons were sequenced at ChunLab, Inc. (Seoul, South Korea) using the Illumina MiSeq platform, according to the manufacturer’s instructions.

### Data analysis using the MiSeq pipeline

The quality of the native sequence was evaluated by FastQC, and low-quality cutoffs for forward and reverse readings were determined. Then I brought forward and reverse readings to QIIME2 (v 2020.2) [47] for quality control, diversity analysis, and sequence classification. The quality control function in DADA2 [48] was used to cut forward, reverse readout and noise cancellation, chimera detection, and removal. Alpha diversity estimates for community abundance included Shannon Index and operational taxonomic unit, and community uniformity estimates included Pielou’s uniformity. Phylogenetic trees were developed in QIIME2 to estimate beta diversity. The pairwise sample estimates (beta diversity) included the Bray–Curtis similarity distance matrix. The classification level of all sample readings was assigned to the species level using the Silva 132 Reference Taxonomy Database (https://docs.qiime2.org/2019.1/data-resources/). Relative proportions were calculated because changes in proportions at the Phlyum level are related to soil conditions. For this analysis, samples were normalized within the group using DESeq2.

We also used linear discriminant analysis effect size (LDA score > 2, *P* value < 0.05) [49] to identify taxa features that were differentially expressed between samples.

### Estimation of viable Firmicutes and Actinobacteria bacteria in soil samples

The ratio of viable Firmicutes and Actinobacteria bacteria to the total number of Gram-negative bacteria in HRS and DRS samples was measured using two methods. A total of 180 bacterial colonies were first tested using the 3% KOH string test [50], and the ratio of Firmicutes and Actinobacteria bacteria to the total number of Gram-negative bacteria was calculated using the following equation:

1 $$	{\mathrm{Viable}}\,{\mathrm{KOH}}\, {\mathrm{reactive}}\,{\mathrm{Gram}}{\mbox{-}}{\mathrm{positive}}\,{\mathrm{bacteria}}\,(\%) \\ 	\quad= \frac{{\mathrm{Number}}\,{\mathrm{of}}\,{\mathrm{non}} \mbox{-} {\mathrm{reactive}}\,{\mathrm{bacterial}}\,{\mathrm{colonies}}}{{\mathrm{Number}}\,{\mathrm{of}}\,{\mathrm{reactive}}\,{\mathrm{bacterial}}\,{\mathrm{colonies}}} \times 100.$$

Then, HRS and DRS microbial fractions were inoculated onto Tryptic Soy Agar (TSA, Difco Laboratories, Detroit, MI, USA) medium containing no selection marker, 20-μg/mL polymyxin B, or 5-μg/mL vancomycin, and were incubated at 30 °C for 1 day. The ratio of Firmicutes and Actinobacteria bacteria to the total number of Gram-negative bacteria was calculated based on the colony-forming unit (CFU) values of bacterial isolates, as shown below:

2 $$	{\mathrm{Viable}}\,{\mathrm{Gram}}\mbox{-}{\mathrm{positive}}\,{\mathrm{bacteria}}\,\left( {\mathrm{\% }} \right) \\ 	\quad= \frac{{{\mathrm{CFU}}\,{\mathrm{on}}\,{\mathrm{TSA}}\,{\mathrm{containing}}\,{\mathrm{polymyxin}}\,{\mathrm{B}}}}{{{\mathrm{CFU}}\,{\mathrm{on}}\,{\mathrm{TSA}}\,{\mathrm{containing}}\,{\mathrm{vancomycin}}}} \times 100.$$

All experiments were performed in triplicate.

### Optimization of vancomycin treatment

To optimize the vancomycin treatment, HRS and DRS microbial fractions (1 g/mL each) were treated with three different concentrations of vancomycin (5, 50, and 500 μg/mL) for three different durations (0, 3, and 6 h). The HRS and DRS fractions treated with 500-μg/mL vancomycin were centrifuged, and pellets were washed twice with 2.5-mM MES buffer to remove residual vancomycin. Soil fractions treated with or without vancomycin were inoculated on TSA medium containing 0- or 5-μg/mL vancomycin. To induce the dysbiosis of Firmicutes and Actinobacteria, each soil fraction was added to 500-μg/mL vancomycin and incubated at 30 °C for 3 h.

To examine the vancomycin sensitivity of the bacterial wilt pathogen and protective bacteria, a suspension (OD_600_ = 1.0) of *R. solanacearum* was plated on a CPG medium containing 2,3,5-triphenyl tetrazolium chloride (TZC), and that of each of the four selected protective bacterial strains HRS1, HRS2, HRS3, and HRS4 was plated on TSA medium. Then, 10-μL vancomycin (500, 50, 5, and 0.5 µg/mL) or kanamycin (50 µg/mL; control) was dropped on each inoculated plate and incubated at 30 °C for 2 days. The plates were examined after 2 days to examine the development of an inhibition zone. All experiments were performed in triplicate.

### Isolation and cultivation of spore-forming bacteria in vitro

To isolate spore-forming bacteria, HRS and DRS fractions were incubated at 80 °C for 30 min [51], plated on TSA medium, and incubated at 30 °C for 48 h. To determine the identity of each bacterial colony, 16S rRNA sequencing was performed at GenoTech (Daejeon, South Korea) using the primer pair 27F/1492R (Table S1). Sequence reads were aligned using BLASTn (http://blast.ncbi.nlm.nih.gov/Blastcgi), and the closest match was identified.

### Root colonization capacity of HRS-specific bacterial isolates

To assess the colonization of tomato roots by the four selected bacterial isolates, CFU values of the four spontaneous rifampicin-resistant strains of HRS1, HRS2, HRS3, and HRS4 were measured as described previously [52, 53]. Briefly, rhizosphere soil suspension and tomato seedling roots treated with each rifampicin-resistant bacterial suspension (OD_600_ = 20) were prepared at 0, 1, and 2 weeks post inoculation (wpi). The rhizosphere soil suspension was incubated on TSA medium containing 100-µg/mL rifampicin for 2–3 days at 30 °C, and then the bacterial population was measured. All experiments were performed in triplicate, with five plants per treatment.

### Evaluation of disease suppression by HRS-specific bacterial isolates

The selected bacterial species were cultured on TSA medium for 24 h and then suspended in sterile distilled water (OD_600_ = 1.0). Tomato seedling roots were washed with sterile water and immersed in each bacterial suspension for 30 min. Then, the tomato seedlings were transplanted in sterilized soil and grown at 28 °C for 5 days. Subsequently, tomato roots were inoculated with *R. solanacearum* via drench application, as described above. This experiment was performed in triplicate, with 12 plants per treatment.

To perform the ISR test, roots of tomato seedlings were treated with the four selected bacterial strains HRS1, HRS2, HRS3, and HRS4 and two soil fractions (HRS and DRS). After 7 days, 50-μL *R. solanacearum* suspension was injected into the stem of these tomato plants using a 200-μL pipette tip. To conduct the SynCom treatment, suspension cultures of all four strains were mixed, and the final OD_600_ of each bacteria was adjusted to 1.0. This experiment was performed in triplicate, with 12 plants per treatment.

Antagonistic effects of each of the four selected isolates against *R. solanacearum* were examined using the co-culture method [54]. A suspension of *R. solanacearum* (OD_600_ = 1.0) was plated on TSA medium. Then, 10 μL each of HRS1, HRS2, HRS3, and HRS4 (OD_600_ = 1.0) or of gentamycin (0.5 mg/mL; control) was dropped on the pathogen-inoculated plate. The plates were then incubated at 30 °C, and the development of an inhibition zone was examined after 2 days. This experiment was performed in triplicate.

### Expression analysis of defense signaling marker genes in tomato

Total RNA was extracted from tomato leaves harvested at 0 and 12 h after pathogen inoculation, and first-strand cDNA was synthesized as described previously [55]. Then, quantitative real-time PCR (qRT-PCR) was performed on the Chromo4 Real-Time PCR System (Bio-Rad, Hercules, CA, USA) using the cDNA template, iQTM SYBR® Green Supermix (Bio-Rad), and 10-pM sequence-specific primers (Table S2) under the following conditions: initial polymerase activation at 95 °C for 10 min, followed by 40 cycles of denaturation at 95 °C for 30 s, annealing at 60 °C for 30 s, and extension at 72 °C for 30 s. The expression level of each gene was calibrated and normalized relative to that of *Ubiquitin3* mRNA. All qRT-PCR experiments were performed in triplicate.

### Statistical analysis

Two-way analysis of variance and two-tailed Student’s *t* test were performed in the R program [56] to analyze the data. Differences were considered statistically significant at *P* < 0.05.

## Results

### Uneven distribution of bacterial wilt symptoms in the greenhouse

**Fig. 1:**
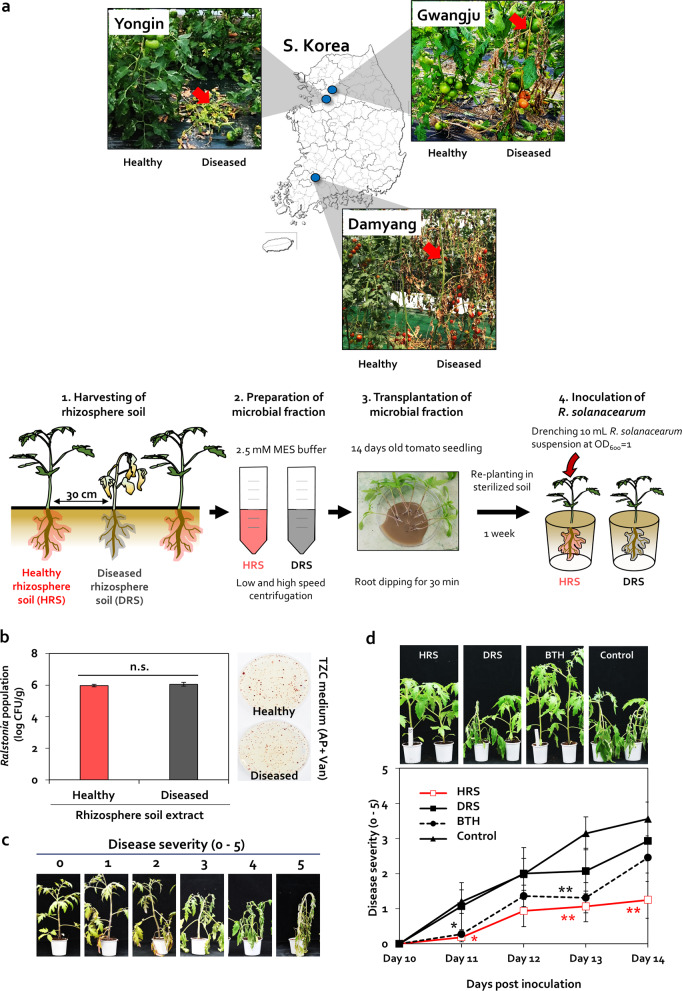
Differences in rhizosphere disease suppression between two adjacent tomato plants. **a** Images of healthy and diseased tomato plants grown within a 30-cm distance in three different locations (Damyang, Yongin, and Gwangju) in South Korea. Red arrows indicate the wilted tomato plants infected by *Ralstonia solanacearum*. To prepare the microbial fraction, healthy rhizosphere soil (HRS) and diseased rhizosphere soil (DRS) were suspended in 2.5-mM MES buffer. Roots of 14-day-old tomato seedlings were dipped in the microbial fractions for 30 min. Severity of bacterial wilt disease caused by *R. solanacearum* was quantified. Data represent mean ± standard error of the mean (SEM; *n* = 12 plants per treatment). Asterisks indicate significant differences (*P < 0.05, **P < 0.01, ***P < 0.001). **b** Cell density of *R. solanacearum* in HRS and DRS fractions plated on casamino acid-peptone-glucose (CPG) agar medium containing 2,3,5-triphenyl tetrazolium chloride (TZC), 0 or 50-g/mL ampicillin (AP), and 0 or 5-g/mL vancomycin (Van). **c** Scoring of disease severity on a 0–5 scale. **d** Disease severity in HRS and DRS fraction-treated tomato plants at 10–14 days post inoculation (dpi). BTH, 0.5-mM benzothiadiazole treated tomato; control, 2.5-mM MES buffer-treated tomato.

In this study, we observed the occurrence of bacterial wilt disease in three different greenhouses located in Yongin, Gwangju, and Damyang, South Korea; however, these symptoms randomly appeared only in local areas (<1-m diameter) and did not spread throughout the greenhouse at the end of season in any of the three locations, where the physicochemical properties of soil differed (Fig. 1a and Table S3). A patch of dead or dying tomato plants affected by bacterial wilt appeared between two healthy plants spaced <30-cm apart (Fig. 1a). The population of the casual pathogen, *R. solanacearum*, was 9.3 × 10^5^ and 1.1 × 10^6^-CFU/g soil in HRS and DRS, respectively, and this difference in population size was not statistically significant (*P* = 0.05) (Fig. 1b). The inoculum potential of *R. solanacearum* at 10^2–3^ CFU/mL is sufficient to cause bacterial wilt [57]. Therefore, changes in the pathogen population in the rhizosphere did not underlie the difference in the occurrence of bacterial wilt between healthy and diseased plants.

Because the composition of rhizosphere microbiome can be used to determine the suppression of bacterial wilt in tomato, as demonstrated previously [4, 58], we examined whether microbial fractions prepared from HRS and DRS samples were responsible for bacterial wilt occurrence (Fig. 1a). In plants treated with 2.5-mM MES buffer (negative control), bacterial wilt symptoms began to appear at 11 dpi (Fig. 1a). Treatment of tomato plants with 0.5-mM benzothiadiazole (BTH; a positive control), which activates plant immunity against *R. solanacearum* without antagonism [59], reduced the disease severity by 75% at 11 dpi and by 58% at 13 dpi, but not at 12 or 14 dpi, compared with the control (Fig. 1d). The HRS fraction significantly reduced bacterial wilt severity by 83%, 65%, and 64% at 11, 13, and 14 dpi, respectively, compared with the control, but did not reduce disease severity at 12 dpi (Fig. 1d). On the other hand, the DRS fraction failed to suppress bacterial wilt disease at all time points (Fig. 1d). These data indicate that the difference in bacterial wilt occurrence between two tomato plants was caused by the difference in soil microbiome composition between HRS and DRS fractions, rather than by the difference in pathogen abundance.

### Comparison of microbiome between HRS and DRS fractions

**Fig. 2:**
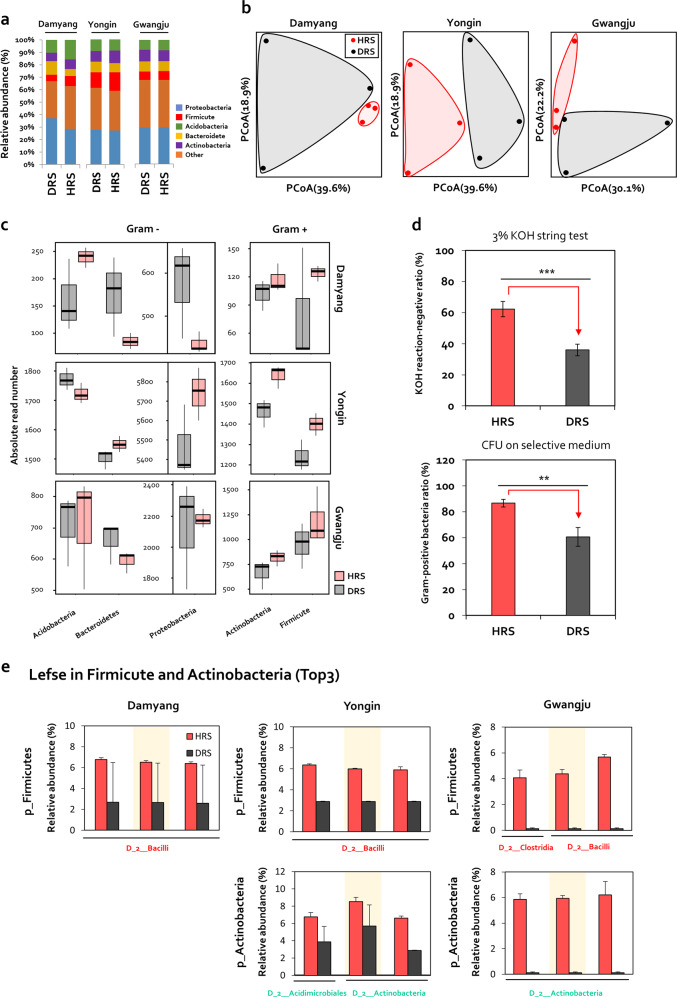
Comparison of soil community structure between HRS and DRS samples based on pyrosequencing of 16S rRNA amplicons. **a** Relative abundance of rhizobacteria at the phylum level in HRS and DRS samples collected from greenhouses in Damyang, Yongin, and Gwangju in South Korea. **b** Two-dimensional principal coordinate analysis (PCoA) of Bray–Curtis dissimilarity. Significant differences in microbial community composition were detected between HRS and DRS samples in Damyang, Yongin, and Gwangju. **c** The absolute read numbers of Firmicute, Actinobacteria, Proteobacteria, Acidobacteria, and Bacteroidetes in HRS and DRS from fields in Damyang, Yongin, and Gwangju. Gram+ Gram-positive bacterial groups, Gram− Gram-negative bacterial groups. **d** Measurement of the ratio of viable Firmicutes and Actinobacteria to Gram-negative bacteria in HRS and DRS samples using the 3% KOH string test and based on the quantification of colony-forming unit (CFU) values of bacterial isolates on TSA medium containing 20-μg/mL polymyxin B (toxic to Gram-negative bacteria) and 5-μg/mL vancomycin (toxic to Gram-positive bacteria). Data represent mean ± SEM. Asterisks indicate significant differences (*P < 0.05, **P < 0.01, ***P < 0.001). **e** LefSe analysis of the Firmicutes and Actinobacteria community in HRS and DRS samples collected from Damyang, Yongin, and Gwangju. LefSe analysis was used to identify the most discriminating ASVs of Firmicutes and Actinobacteria phyla in HRS.

To detect differences in microbial composition between healthy and diseased rhizosphere samples, seven HRS and eight DRS collected from Damyang, Yongin, and Gwangju were subjected to 16S rDNA amplicon sequencing (Fig. 2). Relative abundance analysis indicated that Proteobacteria, Firmicutes, Actinobacteria, Acidobacteria, and Bacteroidete were major bacterial communities at the phylum level (Fig. 2a). Alpha diversity analysis revealed no differences in bacterial evenness and richness indices (Fig. S1), whereas principal coordinate analysis, based on the Bray–Curtis dissimilarity index, revealed clear differences between HRS and DRS samples (Fig. 2b). Among five major phyla, the read numbers of Gram-positive Firmicutes and Actinobacteria were higher in HRS samples than in DRS samples (Fig. 2c). The read numbers of Firmicutes in HRS were increased by 1.57-fold, 1.13-fold, and 1.25-fold in Damyang, Yongin, and Gwangju, respectively (Fig. 2c). The read numbers of Actinobacteria in HRS were increased by 1.15-fold, 1.12-fold, and 1.23-fold in Damyang, Yongin, and Gwangju, respectively (Fig. 2c). On the other hand, the read numbers of Proteobacteria and Bacteroidetes phyla were lower in HRS samples than in DRS samples in Damyang and Gwangju, but higher in Yongin (Fig. 2c). Conversely, the read numbers of Acidobacteria phyla were lower in HRS samples than DRS samples in Yongin, but higher in Damyang and Gwangju (Fig. 2c). To validate the enrichment of viable Firmicutes and Actinobacteria in the HRS fraction, we conducted the 3% KOH string test, and cultured Firmicutes and Actinobacteria on selective media containing 20-μg/mL polymyxin B or 5-μg/mL vancomycin to calculate their CFU values (Fig. 2d). In both experiments, the ratio of viable Firmicutes and Actinobacteria to Gram-negative bacteria in HRS samples was increased by 26.2% and 26.3%, respectively, compared with DRS samples (Fig. 2d).

To explore the most discriminating amplicon sequence variant (ASV) in Firmicutes and Actinobacteria in HRS, we selected the top three Firmicute and Actinobacteria ASVs showing a significant difference in relative abundance between HRS and DRS fractions using the Lefse method (Fig. 2e). Among Firmicute ASVs, three Bacilli class ASVs in Damyang and Yongin, and two Bacilli class ASVs and one Clostridia class ASV in Gwangju, were the most discriminating ASVs enriched in HRS samples (Fig. 2e). Meanwhile, among Actinobacteria ASVs, two Actinobacteria class ASVs and one Acidimicrobiales class ASV in Yongin, and three Actinobacteria class ASVs in Gwangju, were the most discriminating ASVs enriched in HRS samples (Fig. 2e). Collectively, these data led us to hypothesize that changes in the relative abundance of Bacilli and Actinobacteria classes in the rhizosphere determine the suppression of bacterial wilt in tomato.

### Effect of Firmicutes and Actinobacteria disruption on disease suppression in HRS

**Fig. 3:**
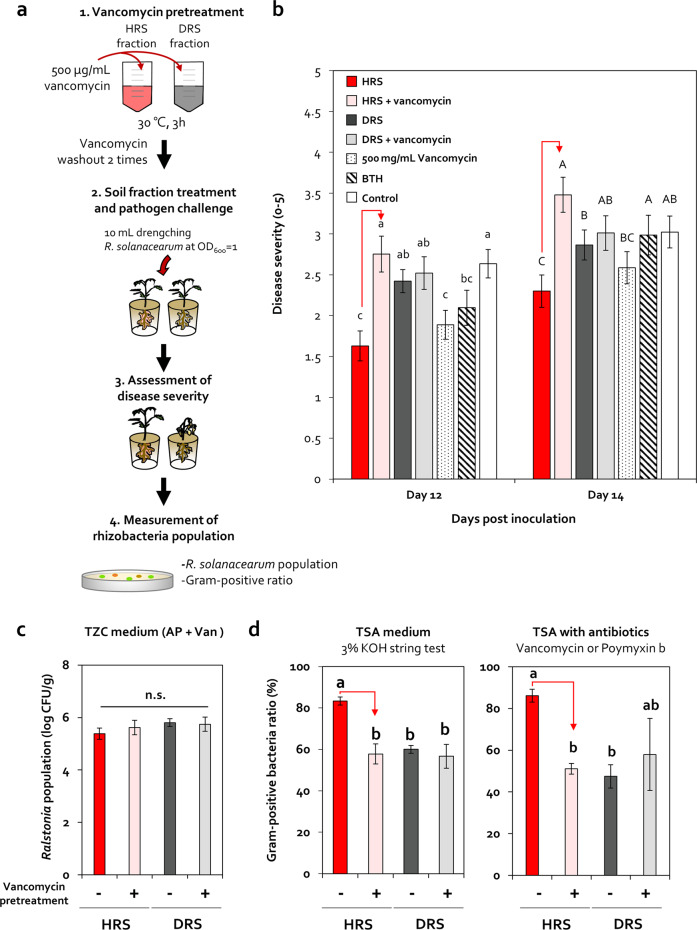
Dysbiosis of Firmicutes and Actinobacteria in the tomato rhizosphere. **a** Disease severity and viable Firmicutes ratio in HRS and DRS samples pretreated with or without 500-μg/mL vancomycin. After 3-h incubation at 30 °C, soil fractions were washed twice with 2.5-mM MES buffer. The prepared soil fractions pretreated with or without vancomycin were applied to tomato roots via the root-dipping method. **b** Severity of bacterial wilt disease in tomato plants treated with HRS and DRS fractions, with or without 500-μg/mL vancomycin pretreatment. HRS HRS fraction, HRS + vancomycin HRS pretreated with 500-µg/mL vancomycin, DRS DRS fraction, DRS + vancomycin DRS pretreated with 500-µg/mL vancomycin, 500-mg/mL vancomycin root-dipping treatment with 500 mg/mL vancomycin. Data represent mean ± SEM. Different letters indicate significant differences between treatments (*P* < 0.05; least significant difference [LSD] test). **c** Changes in *R. solanacearum* cell density in HRS and DRS fractions pretreated with or without 500-μg/mL vancomycin. **d** Changes in Firmicutes abundance in HRS and DRS fractions pretreated with or without 500-μg/mL vancomycin. The ratio of viable Firmicutes bacteria was measured using the 3% KOH string test and by the quantification of the CFU values of bacterial isolates grown on TSA medium containing 20-μg/mL polymyxin B or 5-μg/mL vancomycin.

To examine the role of Firmicutes and Actinobacteria in bacterial wilt incidence in tomato, we specifically inhibited the growth of Firmicutes and Actinobacteria in HRS using vancomycin, which is an antibiotic against Gram-positive bacteria [60] (Figs. 3a and S2). Based on the optimization experiment (Fig. S2 and Table 1), HRS and DRS fractions pretreated with or without 500-μg/mL vancomycin were applied to the tomato root system (Figs. 3b and S3). Compared with the HRS treatment (HRS), vancomycin-pretreated HRS (HRS + vancomycin) significantly increased bacterial wilt severity by 1.8-, 1.7-, 1.5-, 1.5-, and 1.5-fold at 12, 13, 14, 15, and 16 dpi, respectively (Figs. 3b and S3). Conversely, vancomycin-pretreated DRS (DRS + vancomycin) did not alter bacterial wilt severity compared with the DRS treatment (DRS) (Figs. 3b and S3). Compared with the control, exogenous vancomycin treatment (500 mg/mL) did not reduce the severity of bacterial wilt (Fig. 3b and S3), and a droplet of vancomycin (0.5, 5, 50, and 500 µg/mL) did not directly inhibit the growth of *R. solanacearum* (Fig. S4a).

**Table 1:** Bacterial population in vancomycin-pretreated healthy rhizosphere soil samples grown on TSA medium containing or lacking 5-µg/mL vancomycin.

Vancomycin concentration in soil extract (µg/mL)	Bacterial population (log CFU/g)								
	0-h incubation			3-h incubation			6-h incubation		
	w/ vancomycin	w/o vancomycin	*p* value	w/ vancomycin	w/o vancomycin	*P* value	w/ vancomycin	w/o vancomycin	*P* value
0	5.431 ± 0.159	6.113 ± 0.034	0.014	5.489 ± 0.093	6.111 ± 0.073	0.006	5.648 ± 0.117	6.130 ± 0.081	0.028
5	5.433 ± 0.124	6.073 ± 0.051	0.009	5.306 ± 0.073	6.015 ± 0.032	0.001	5.621 ± 0.130	6.257 ± 0.043	0.010
50	5.463 ± 0.108	6.042 ± 0.074	0.011	5.494 ± 0.100	6.991 ± 0.116	0.032	5.648 ± 0.174	6.077 ± 0.034	0.073
500	5.358 ± 0.086	6.004 ± 0.149	0.020	5.385 ± 0.089	5.442 ± 0.087	0.673	5.380 ± 0.123	5.636 ± 0.098	0.180

w/ = with, w/o = without.

*CFU* colony-forming units, *w/ vancomycin* TSA medium containing 5-µg/mL vancomycin; *w/o vancomycin* TSA medium lacking vancomycin.

Interestingly, the viable Firmicutes and Actinobacteria ratio in HRS + vancomycin-treated tomato rhizosphere was significantly lower than that in HRS-treated tomato rhizosphere by 25.5% in the 3% KOH string test, and by 35.1% on selective medium (based on CFU values) at 5 wpi (Fig. 3d); by contrast, no differences were detected between DRS and DRS + vancomycin treatments (Fig. 3d). However, *R. solanacearum* and total bacterial population showed no significant differences between HRS, HRS + vancomycin, DRS, and DRS + vancomycin treatments at 5 wpi (Figs. 3c and S5). These data indicate that disruption of vancomycin-sensitive HRS-specific Firmicutes and Actinobacteria attenuated HRS-mediated disease suppression against *R. solanacearum*.

### Isolation of Firmicutes and Actinobacteria enriched in HRS samples

**Fig. 4:**
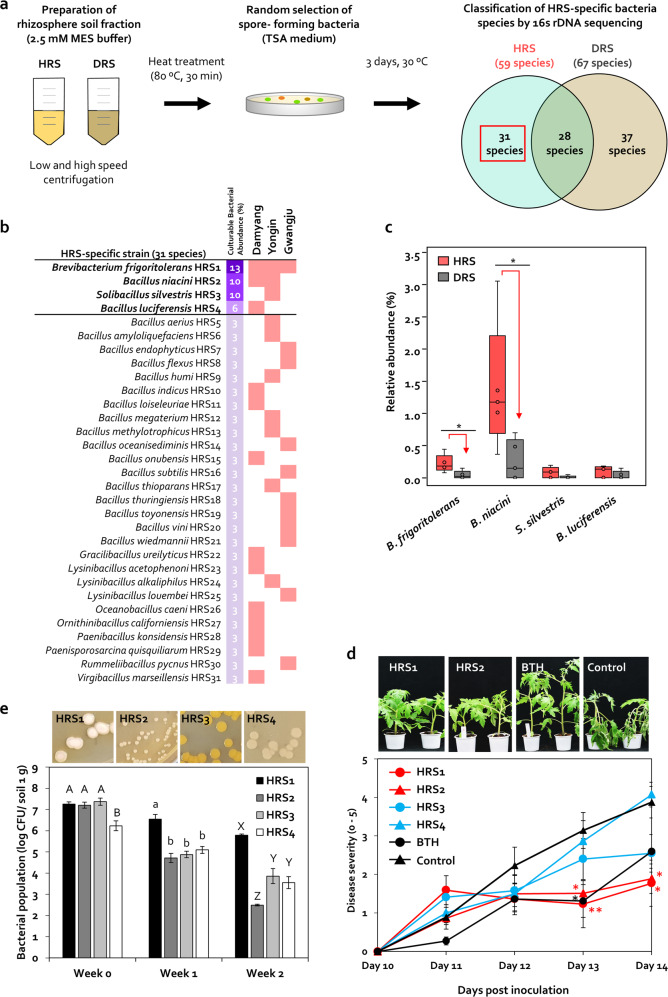
Identification of heat-stable Firmicutes and Actinobacteria in HRS and DRS samples. **a** Isolation of spore-forming Firmicutes bacteria from HRS and DRS fractions treated with high temperature (80 °C) for 30 min. Heat-treated soil fractions were inoculated on TSA medium and incubated at 30 °C for 3 days. **b** Identification of HRS-specific Firmicutes bacteria present in soil from tomato fields in Damyang, Yongin, and Gwangju. A total of 326 colonies of spore-forming bacteria were randomly selected and identified by 16S rDNA sequencing, and 21 species were identified as HRS-specific bacteria. **c** Investigation of the distribution of selected bacteria in the tomato rhizosphere using 16S rRNA sequencing. **d** Severity of bacterial wilt in tomato seedlings treated with HRS-specific Firmicutes bacteria. Data represent mean ± SEM (*n* = 12 plants per treatment). Asterisks indicate significant differences (*P < 0.05, **P < 0.01, ***P < 0.001).

Out of 326 bacterial colonies, 59 and 67 Gram-positive bacteria belonging to Firmicutes and Actinobacteria phyla were isolated from heat-treated HRS and DRS fractions, respectively (Fig. 4a), and 30 Bacillales and one Actinomycetales were specifically isolated from three different HRS samples (Fig. 4a, b). One Actinomycetales (*Brevibacterium frigoritolerans*) and 12 Bacillales (*Bacillus niacini*, *B. luciferensis*, *B. indicus, B. loiseleuriae, B. onubensis, Gracilibacillus ureilyticus, Lysinibacillus acetophenoni, Oceanobacillus caeni, Ornithinibacillus californiensis, Paenibacillus konsidensis, Paenisporosarcina quisquiliarum*, and *Virgibacillus marseillensis*) were isolated from the Damyang HRS sample (Fig. 4b). One Actinomycetales (*B. frigoritolerans*) and nine Bacillales (*B. niacini*, *Solibacillus silvestris*, *B. aerius*, *B. amyloliquefaciens*, *B. humi*, *B. megaterium*, *B. methylotrophicus*, *B. thioparans*, and *L. alkaliphilus*) were isolated from the Yongin HRS sample (Fig. 4b). One Actinomycetales (*B. frigoritolerans*) and ten Bacillales (*B. endophyticus*, *B. flexus*, *B. oceanisediminis, B. subtilis, B. thuringiensis, B. toyonensis, B. vini, B. wiedmannii, L. louembei*, and *Rummeliibacillus pycnus*) were isolated from the Gwangju HRS sample (Fig. 4b). *B. frigoritolerans* HRS1 (HRS1), *B. niacini* HRS2 (HRS2), *S. silvestris* HRS3 (HRS3), and *B. luciferensis* HRS4 (HRS4) were selected as keystone taxa isolates conferring HRS, with culturable bacterial abundance >5% (13%, 10%, 10%, and 6%, respectively) (Fig. 4b). HRS1 was isolated from all three regions, HRS2 was isolated from Damyang and Yongin, while HRS3 and HRS4 were specifically isolated only from Yongin and Damyang, respectively (Fig. 4b). These four isolates were sensitive to 500-μg/mL vancomycin (Fig. S4b). The relative abundance of *B. frigoritolerans* and *B. niacini* in HRS fractions was 5.5- and 5.22-fold higher than that in DRS fractions, respectively (Fig. 4c and Table 2). Although the relative abundance of *S. silvestris* and *B. luciferensis* in HRS was slightly higher than that in DRS, this difference was not statistically significant (Fig. 4c).

**Table 2:** Relative abundance of healthy rhizosphere soil-specific bacteria in the tomato rhizosphere.

# Fields: query acc.ver	Subject acc.ver	Identity (%)	Alignment length	Mismatches	Gaps	Query start	Query end	Subject start	Subject end	*E* value	Bit score
*Brevibacterium frigoritolerans*	125	98.434	511	3	5	31	537	513	4	0	894
*Bacillus niacini*	3843	98.778	491	4	2	47	537	492	4	0	872
*Solibacillus silvestris*	135	99.785	465	1	0	674	1138	465	1	0	854
*Bacillus luciferensis*	136	95.923	466	18	1	345	810	1	465	0	754

Compared with the control, strains HRS1 and HRS2 significantly reduced disease severity at 13 dpi (by up to 2.6- and 2.1-fold, respectively) and 14 dpi (by 2.2- and 2.1-fold, respectively) (Fig. 4d). By contrast, BTH reduced disease severity only at 13 dpi (by 2.4-fold), while HRS3 and HRS4 failed to suppress bacterial wilt disease at all time points (Fig. 4d). These results indicate that *B. frigoritolerans* (HRS1) and *B. niacini* (HRS2) play an important role in bacterial wilt suppression in tomato.

### Population dynamics of the introduced rhizosphere Firmicutes and Actinobacteria

The population of HRS1, HRS2, HRS3, and HRS4 was 1.8 × 10^7^, 1.6 × 10^7^, 2.4 × 10^7^, and 1.7 × 10^6-^CFU/g soil, respectively, at 0 wpi; 3.5 × 10^6^, 5.2 × 10^4^, 7.6 × 10^4^, and 1.3 × 10^5^-CFU/g soil, respectively, at 1 wpi; and 6.1 × 10^5^, 3.1 × 10^2^, 7.3 × 10^3^, and 3.6 × 10^3^-CFU/g soil, respectively, at 2 wpi (Fig. 4e). However, these four strains were not detected in macerated surface-sterilized root and stem tissues at any time point (data not shown).

### Activation of plant immunity by HRS and individual rhizobacteria

**Fig. 5:**
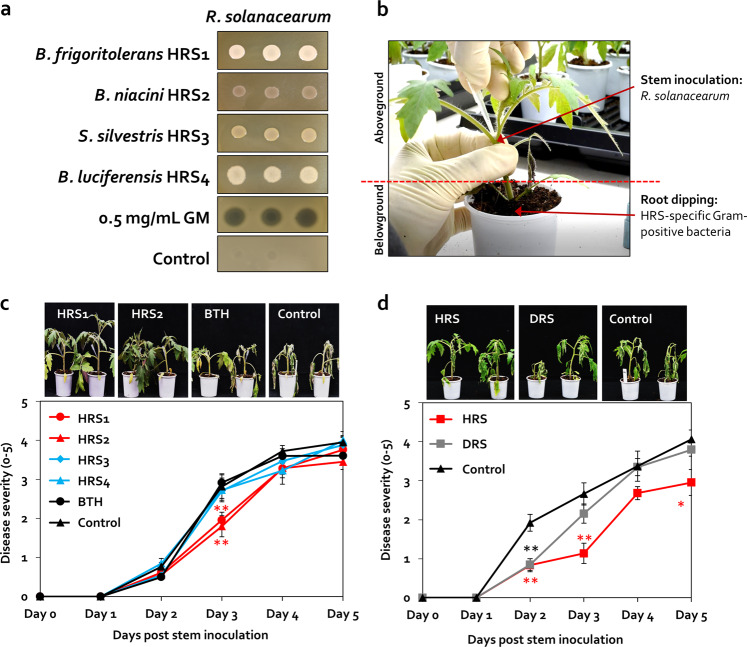
Activation of induced systemic resistance (ISR) against *Ralstonia solanacearum* in HRS samples by the spore-forming Firmicutes and Actinobacteria. **a** Co-cultivation of HRS-specific Firmicutes bacteria and bacterial wilt pathogen *R. solanacearum* on TSA agar medium. Four selected bacterial strains (50 μL; OD_600_ = 1), gentamycin (GM; 0.5 mg/mL; positive control), or sterile distilled water (Control; negative control) were dispensed on a lawn of *R. solanacearum* on TSA agar plates, and photographs were captured after 2 days. **b** Spatial separation system. A suspension of *R. solanacearum* (50 µL; OD_600_ = 1) was injected into the tomato stem 7 days after the root system was treated with each of the four selected Firmicutes strains. Severity of bacterial wilt disease caused by the injection of *R. solanacearum* suspension into the stems of tomato plants treated with HRS-specific Firmicutes bacteria (**c**), or with HRS and DRS fractions (**d**). Data represent mean ± SEM (*n* = 12 plants per treatment). Asterisks indicate significant differences (*P < 0.05, **P < 0.01, ***P < 0.001).

Because of the lack of antagonism between *R. solanacearum* and individual strains during co-cultivation (Fig. 5a), we hypothesized that the four rhizobacterial strains suppress *R. solanacearum* by activating ISR in tomato. While the four isolates were applied to the tomato root, *R. solanacearum* was injected into the stem of the tomato plant to maintain spatial separation between the rhizobacteria and the pathogen [61, 62] (Fig. 5a, b).

In the control treatment, bacterial wilt symptoms developed 9 days earlier with stem inoculation compared with root drench application (Fig. 5c, d). BTH significantly reduced disease severity by 1.5-fold at 2 dpi compared with the control (Fig. 5c). HRS1 and HRS2 reduced disease severity by 1.4- and 1.6-fold, respectively, at 3 dpi compared with control, and their effect on disease suppression was >1.5-fold greater than the effect of BTH (Fig. 5c). However, HRS3 and HRS4 failed to suppress disease severity (Fig. 5c). These data show that HRS1 and HRS2 elicit ISR against *R. solanacearum* in tomato. In addition, ISR activated by the HRS fraction was twofold greater than that activated by HRS1 or HRS2 at 3 dpi, and was maintained until 5 dpi (Fig. 5d). The HRS fraction significantly reduced disease severity by 2.4-, 2.5-, and 1.4-fold at 2, 3, and 5 dpi, respectively, compared with the control (Fig. 5d). However, the DRS fraction failed to suppress bacterial wilt disease, except at 2 dpi (Fig. 5d). These results led us to hypothesize that Firmicutes and Actinobacteria elicit a combinatorial effect in HRS.

### Activation of ISR by a minimum SynCom of Firmicutes and Actinobacteria

**Fig. 6:**
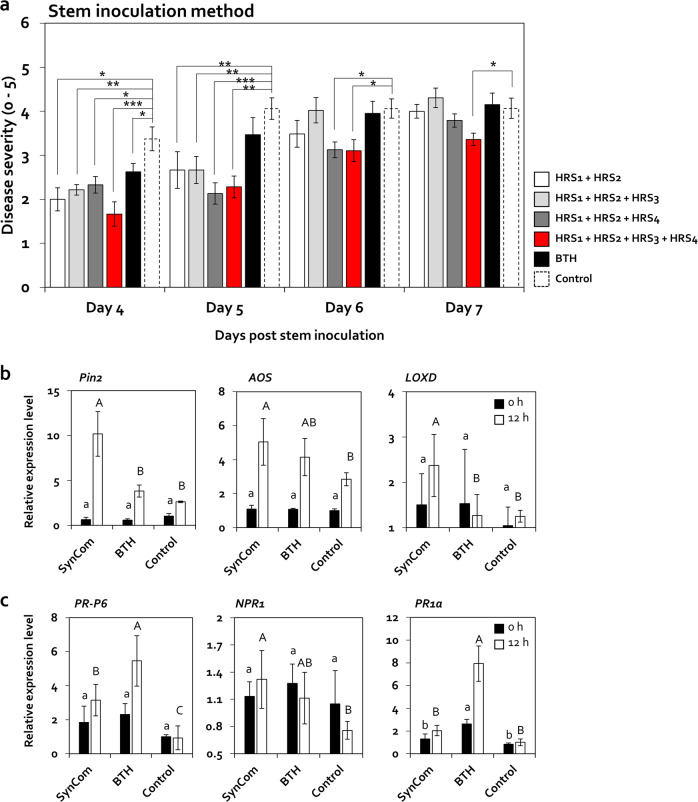
Activation of ISR by a SynCom comprising four HRS-specific Firmicutes strains (HRS1–4). **a** Severity of bacterial wilt disease in tomato plants inoculated with *Ralstonia solanacearum* after treatment with different combinations of the four selected Firmicutes bacteria. HRS1 + HRS2 mixture of *Brevibacterium frigoritolerans* (HRS1) and *Bacillus niacini* (HRS2), HRS1 + HRS2 + HRS3 mixture of HRS1, HRS2, and *Solibacillus silvestris* (HRS3), HRS1 + HRS2 + HRS4 mixture of HRS1, HRS2, and *Bacillus luciferensis* (HRS4), HRS1 + HRS2 + HRS3 + HRS4 mixture of all four Firmicutes strains. Data represent mean ± SEM (*n* = 12 plants per treatment). Asterisks indicate significant differences (*P < 0.05, **P < 0.01, ***P < 0.001). **b, c** Relative expression levels of jasmonic acid (JA) signaling marker genes (**b**) and salicylic acid (SA) signaling marker genes (**c**) in systemic leaves of tomato plants treated with the SynCom comprising all four HRS-specific strains (HRS1–4) at 0 and 12-h post inoculation (hpi) with *R. solanacearum*. Different letters indicate significant differences between treatments (*P* < 0.05; LSD test). Data represent mean ± SEM. SynCom mixture of all four Firmicutes bacterial strains, BTH 0.5-mM BTH treatment, control 2.5-mM MES buffer treatment.

HRS1 and HRS2 failed to reduce bacterial wilt disease after 4 dpi when applied individually (Fig. 5c); however, a mixture of these two strains (HRS1 + HRS2) significantly reduced disease severity by 1.7- and 1.5-fold at 4 and 5 dpi, respectively, compared with the control, and to similar levels as the HRS fraction (Figs. 5d and 6a). Therefore, we generated a minimum SynCom by amending the two-strain mixture (HRS1 + HRS2) with HRS3 and/or HRS4, and tested its effect on disease severity (Fig. 6a). Although the HRS1 + HRS2 + HRS3 mixture reduced bacterial wilt severity by 1.5-fold at both 4 and 5 dpi compared with the control, it showed no significant difference compared with HRS1 + HRS2 (Fig. 6a). On the other hand, the HRS1 + HRS2 + HR4 mixture reduced disease severity by 1.5-, 2.0-, and 1.3-fold at 4, 5, and 6 dpi, respectively, compared with the control (Fig. 6a). A mixture of all four isolates (HRS1 + HRS2 + HRS3 + HRS4) further reduced disease severity by 2.0-, 1.8-, 1.3-, and 1.2-fold at 4, 5, 6, and 7 dpi, respectively, compared with the control (Fig. 6a). These data indicate that a SynCom comprising all four isolates activated the highest level of ISR against *R. solanacearum* in tomato.

To examine whether the SynCom comprising all four bacterial isolates activates defense signaling in tomato, we analyzed the expression patterns of the defense-related marker genes involved in JA, SA, ET, and abscisic acid (ABA) signaling in systemic leaves at 0 and 12-h post inoculation (hpi) (Figs. 6b and S6). Treatment with the SynCom upregulated the expression of JA signaling marker genes *Pin2*, *AOS*, and *LoxD* by 3.9-, 1.8-, and 1.9-fold, respectively, at 12 hpi compared with the control; however, treatment with BTH did not activate these genes (Fig. 6b). In addition, compared with the control, SynCom upregulated the expression of SA signaling marker genes *PR-P6*, *NPR1*, and *PR1a* by 3.5-, 1.7-, and 2.0-fold, respectively, at 12 hpi (Fig. 6b), whereas BTH activated these genes by 6.1-, 1.5-, and 7.9-fold, respectively (Fig. 6b). However, the expression of ET and ABA signaling genes was not activated by the SynCom or BTH (Fig. S6). These data showed that the SynCom primed JA- and SA-dependent ISR against *R. solanacearum* in tomato.

## Discussion

Plants manipulate rhizosphere microbiota to establish disease suppression in the soil [2–4, 63]. Recent studies mostly focused on disease suppression by protective single bacteria and unidentified bacterial consortium in the rhizosphere [3, 16, 32, 64–66]. In animal science, dysbiosis of protective microbiota has been correlated with disease incidence[37–41, 67]; however, in plants, the effect of the disruption of rhizosphere bacteria on disease suppression is largely unknown. In this study, we showed that disruption of ISR-eliciting Firmicutes and Actinobacteria abundance in tomato rhizosphere conferred suppression of bacterial wilt (Figs. 1e and 2) [40, 41, 67]. Because a homeostatic balance of microbial community composition is important for healthy host–microbe relationships, both the enrichment and disruption of microbiota abundance serve as important mechanisms of disease incidence in plants [67–71].

To confirm the role of rhizosphere microbiota disruption in disease suppression, we used vancomycin to disrupt populations of bacteria belonging to Firmicutes and Actinobacteria phyla in tomato rhizosphere. Previous studies employed a pasteurization method, involving the use of moist heat, methyl bromide, or chloropicrin, which kills a wide range of microbes in the soil [6]; therefore, it was difficult to disrupt specific taxa in these studies. On the other hand, vancomycin specifically inhibits cell wall biosynthesis in Firmicutes and Actinobacteria [72]. Therefore, in this study, vancomycin pretreatment reduced the population of Firmicutes and Actinobacteria, and increased bacterial wilt occurrence in only HRS, not in DRS, without changing pathogen abundance (Fig. 3). This result suggests that the disruption of HRS-specific vancomycin-sensitive Firmicutes and Actinobacteria taxa in the rhizosphere plays a critical role in disease suppression.

Intriguingly, *R. solanacearum* reduced the diversity and abundance of non-pathogenic rhizobacteria [58]; however, the population size of *R. solanacearum* was similar in HRS and DRS fractions (Fig. 1d). This raised a fundamental question: what are the driving forces that cause disruption of Firmicutes and Actinobacteria in DRS? First, it is possible that root exudates vary between HRS and DRS. Host plant-derived root exudates including SA, JA, 6-methoxy-benzoxazolin-2-one, amino acids, and organic acids reshape the rhizosphere microbiota and modulate plant immunity [2, 63, 73]. In our previous study, the bacterial volatile 2,3-butanediol induced the secretion of root exudates, which selectively inhibited the growth of specific rhizobacteria in pepper [36]. Root exudates of *Arabidopsis* plants containing an antifungal compound, scopoletin, also selectively suppressed the growth of fungal pathogens, whereas the beneficial plant growth-promoting rhizobacteria were resistant to scopoletin, which is more effective against Gram-positive bacteria than Gram-negative bacteria [60, 74]. Further profiling of antibacterial compounds in root exudates should be conducted using liquid chromatography–mass spectrometry or gas chromatography–MS analyses. In addition, comparative genomic analysis of tomato plants with HRS and DRS phenotypes grown in HRS and DRS is also required because root exudate composition varies with the host genotype [74–76].

The second possible scenario underlying microbial disruption in DRS is the induction of plant immune signaling. The correlation between defense signaling and rhizosphere microbial composition has been studied previously. In *Arabidopsis*, the relative abundance of Firmicutes was lower in the rhizosphere of mutant plants, with reduced immune response, than in the rhizosphere of the wild type [2], and deletion of SA or JA signaling genes, which regulate plant immunity, changed the rhizosphere microbiota [2, 63]. For example, the population of Firmicutes and Actinobacteria genera *Bacillus* and *Streptomyces* was higher in the *med25* mutant rhizosphere than in the wild-type rhizosphere [63]. In addition, in tomato, because host disease resistance changes the rhizosphere microbiota [4], genetic variation in defense signaling between tomato genotypes grown in HRS and DRS can lead to compositional changes in the tomato rhizosphere.

A previous study showed that Firmicutes taxa, *Bacillus* and *Paenibacillus*, and the Actinobacteria taxon, *Streptomyces*, establish disease suppression by a single strain or SynCom via antagonistic effect [6]; however, our data showed JA-dependent ISR activation by SynCom without antagonistic activity (Fig. 6a, b). While SynCom-mediated ISR is largely unknown, a single *Bacillus* strain has been shown to elicit JA-dependent ISR [20, 55, 77–80]. The designed SynCom activated greater ISR against *R. solanacearum* than its constituent individual strains (Figs. 5c and 6a). Similarly, a combination of beneficial rhizobacteria improved ISR and plant immune responses, such as activation of peroxidase, chitinase enzyme, and polyphenol oxidase, compared with individual rhizobacteria [3, 81–84]. The keysonte taxa strains, HRS1 and HRS2, as well as minor strains, HRS3 and HR4, orchestrately played important roles in enhancing ISR against *R. solanacearum*. The relative abundance of Firmicutes taxa was lower in the Gwangju HRS sample (lacking HRS3 and HRS4 strains) than in the Damyang and Yongin HRS samples (Fig. 2a). Recent studies reported that the presence or absence of a specific strain with <0.1% abundance can alter the abundance of other strains in the rhizosphere [85, 86]. Thus, the low-abundant HRS3 and HRS4 strains might boost ISR by the enrichment of Firmicutes and Actinobacteria in the rhizosphere (Figs. 4c and 6). The production of secondary metabolites in SynCom can also enhance ISR activation. *Brevibacterium* produces phenazine, which is not only an antibiotic but also triggers ISR [87, 88]. A mixed culture of *B. subtilis* and *Lactobacillus sakei* increased the production of γ-aminobutyric acid, which triggers the plant immune response [89, 90]. In addition, co-cultivation of *Streptomyces coelicolor* (Actinobacteria) and *B. subtilis* (Firmicutes) increased the production of undecylprodigiosin, which suppressed the fungal pathogen, *Verticillium dahliae* [91, 92]. However, SynCom-derived molecular determinants are likely very complex and should be investigated further.

In this study, we first demonstrated that the specific disruption of the protective Firmicutes and Actinobacteria community in tomato rhizosphere enhanced the incidence of bacterial wilt disease. Although sustained monoculture can lead to a considerable decline in rhizosphere microbiota diversity and consequently local disruption of disease suppression, this diversity can be recovered by amending the soil with a minimal SynCom. Further investigations are needed to (1) identify the factors responsible for the local collapse of disease suppression, (2) identify an early diagnostic marker of microbiota disruption by microbiome analysis before disease occurrence, and (3) understand individual microbial determinants that activate plant immunity. Because of the high stress tolerance of *R. solanacearum* in soil and the limitation of the control method, it is difficult to control the incidence and spread of bacterial wilt [93]. Our results suggest that the emergence of DRS indicates the conversion of disease-suppressive soil into disease-conducive soil. The introduction of SynCom, as a probiotic and prebiotic material that enhances Firmicutes and Actinobacteria abundance, could be a novel and stable biological control method against *R. solanacearum*.

## Supplementary information

Supplementary Table

Supplementary Figures
