# Supplementary material for: Disruption of Firmicutes and Actinobacteria abundance in tomato rhizosphere causes the incidence of bacterial wilt disease
Source: ISME J. 2020 Oct 7;15(1):330–47. doi: 10.1038/s41396-020-00785-x (PMC7852523; doi:10.1038/s41396-020-00785-x)
Supplement: Supplementary file 1 — Supplementary Table [file 41396_2020_785_MOESM1_ESM.docx]

**Table S1. List of primers used for microbiome analysis.**

| **Primer name** | **Primer sequence (5'->3')^†^** |
| --- | --- |
| 341F | TCGTCGGCAGCGTCAGATGTGTATAAGAGACAG-CCTACGGGNGGCWGCAG |
| 805R | GTCTCGTGGGCTCGG-AGATGTGTATAAGAGACAG-GACTACHVGGGTATCTAATCC |
| i5-F | AATGATACGGCGACCACCGAGATCTACAC-XXXXXXXX-TCGTCGGCAGCGTC |
| i7-R | CAAGCAGAAGACGGCATACGAGAT-XXXXXXXX-GTCTCGTGGGCTCGG |
| 27F | AGAGTTTGATCCTGGCTCAG |
| 1492R | GGTTACCTTGTTACGACTT |

^†^X indicates the barcode region.

**Table S2. List of primers used for quantitative real-time PCR (qRT-PCR).**

| **Primer name** | **Primer sequence (5'->3')** |
| --- | --- |
| *Ubi3*-F | GTGTGGGCTCACCTACGTTT |
| *Ubi3*-R | ACAATCCCAAGGGTTGTCAC |
| *Pin2*-F | TGATGCCAAGGCTTGTACTAGAGA |
| *Pin2*-R | AGCGGACTTCCTTCTGAACGT |
| *AOS*_F | CCGGCGGGAAGATCACGATG |
| *AOS*_R | TCGAAAACGGCGTCGTGTGA |
| *LOXD*_F | GCAGTACCGGACGCAACACA |
| *LOXD*_R | CTGCAAACTTGGGCCGAGGA |
| *PR-P6-*F | GTACTGCATCTTCTTGTTTCCA |
| *PR-P6*-R | TAGATAAGTGCTTGATGTGCC |
| *NPR1*-F | TGATGGCACGTCTGAATTACCC |
| *NPR1*-R | AACCGTTTTCCAAGTTCCACAGTT |
| *PR-1a*-F | CACATTTTTCCACCAACACATTG |
| *PR-1a*-R | GAGGGCAGCCGTGCAA |
| *EIN2*-F | GTTGCTAAGTGATGCTGTA |
| *EIN2*-R | CGCTCAAGCATGCTGGGCC |
| *ERF1*-F | TGGAGTTAGAAAGAGGCCATGG |
| *ERF1*-R | CCCTCATTGATAATGCGGCTT |
| *CHI9*-F | GTCATCACCGGAAGATGGCAGC |
| *CHI9*-R | CCGATCCTGGACCCTGCTGT |
| *ABAOH*-F | TTGCTGCACAAGATACAACAGCAAG |
| *ABAOH*-R | TGTCCATGTCAACCCATGATTTTCT |
| *AREB*-F | GCTCAACAGGAGGGAGTGG |
| *AREB*-R | CATCAACAGTCTTATGACTCAG |
| *NCED1*-F | TCGAAAACCCGGATGAACAAGTGA |
| *NCED1*-R | AACCAGAAACTTTTGGCCATGGTTC |

**Table S3. Physicochemical properties of field soil from Damyang, Yongin, and Gwangju.**

| Contents | Damyang | Yongin | Gwangju |
| --- | --- | --- | --- |
| Sand (%) | 52.7 | 40.2 | 54.7 |
| Silt (%) | 34.6 | 42.6 | 35.9 |
| Clay (%) | 12.8 | 17.2 | 10.0 |
| pH | 5.5 ± 0.4 | 6.1 ± 0.1 | 6.1 ± 0.78 |
| Dissolved organic carbon (g/kg) | 16.7 ± 4.1 | 37.2 ± 3.3 | 14.3 ± 3.5 |
| Available phosphate (mg/kg) | 998.7 ± 90.7 | 1712.6 ± 249.3 | 428.7 ± 190.9 |
| Potassium (cmol^+^/kg) | 1.9 ± 0.4 | 3.0 ± 1.0 | 1.2 ± 0.8 |
| Calcium (cmol^+^/kg) | 7.0 ± 2.1 | 10.6 ± 2.6 | 7.0 ± 3.4 |
| Magnesium (cmol^+^/kg) | 1.8 ± 0.6 | 4.4 ± 1.3 | 2.8 ± 1.8 |
| Electrical conductivity (ds/m) | 3.8 ± 2.8 | 10.8 ± 4.2 | 3.6 ± 3.2 |
