# Supplementary material for: Disruption of Firmicutes and Actinobacteria abundance in tomato rhizosphere causes the incidence of bacterial wilt disease
Source: ISME J. 2020 Oct 7;15(1):330–47. doi: 10.1038/s41396-020-00785-x (PMC7852523; doi:10.1038/s41396-020-00785-x)
Supplement: Supplementary file 2 — Supplementary Figures [file 41396_2020_785_MOESM2_ESM.docx]

**Supplementary Information**


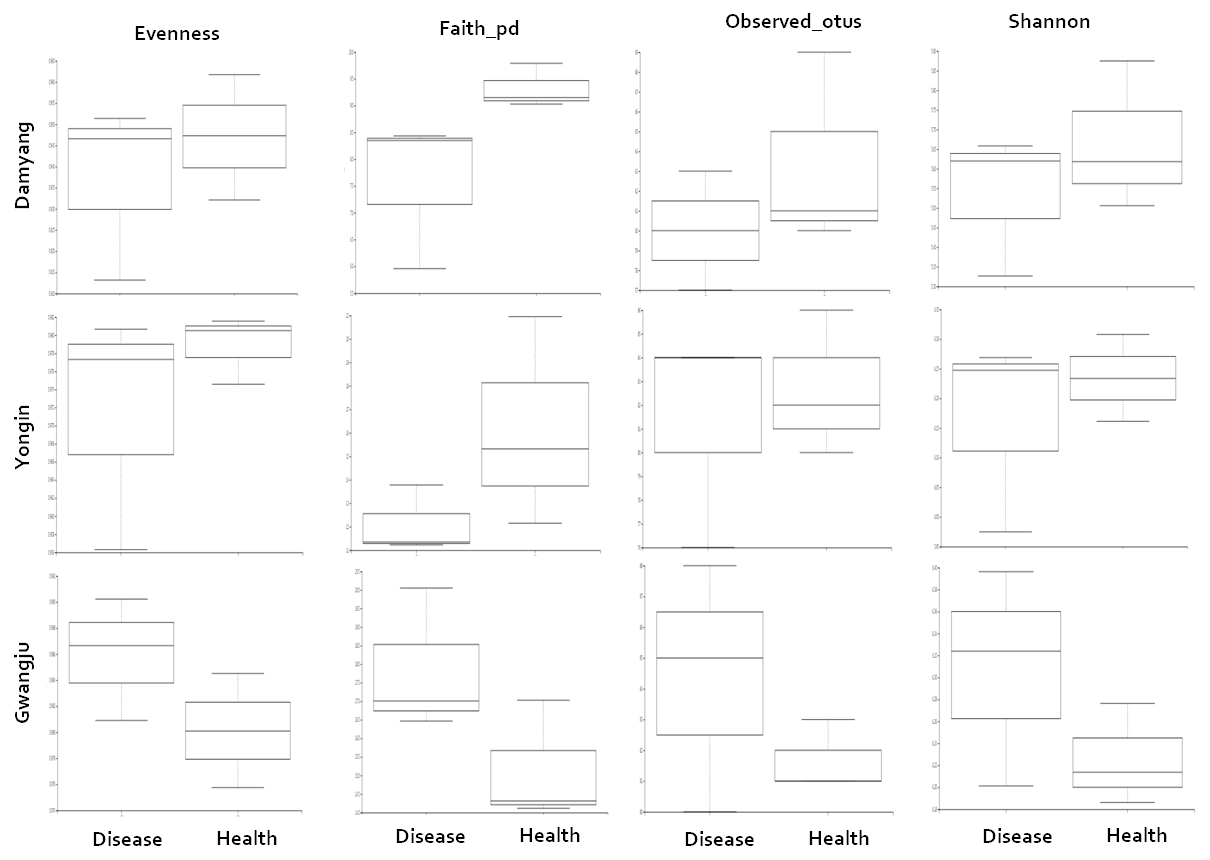


**Figure S1.** Comparison of the evenness and richness of bacteria in healthy rhizosphere soil (HRS) and diseased rhizosphere soil (DRS) of tomato plants using alpha diversity indices. Alpha diversity analysis of HRS and DRS samples collected from Damyang, Yongin, and Gwangju in South Korea. Faith_pd and Observed_otus refer to the richness of species, whereas Shannon and Evenness indices indicate the evenness of species.

**a**

**
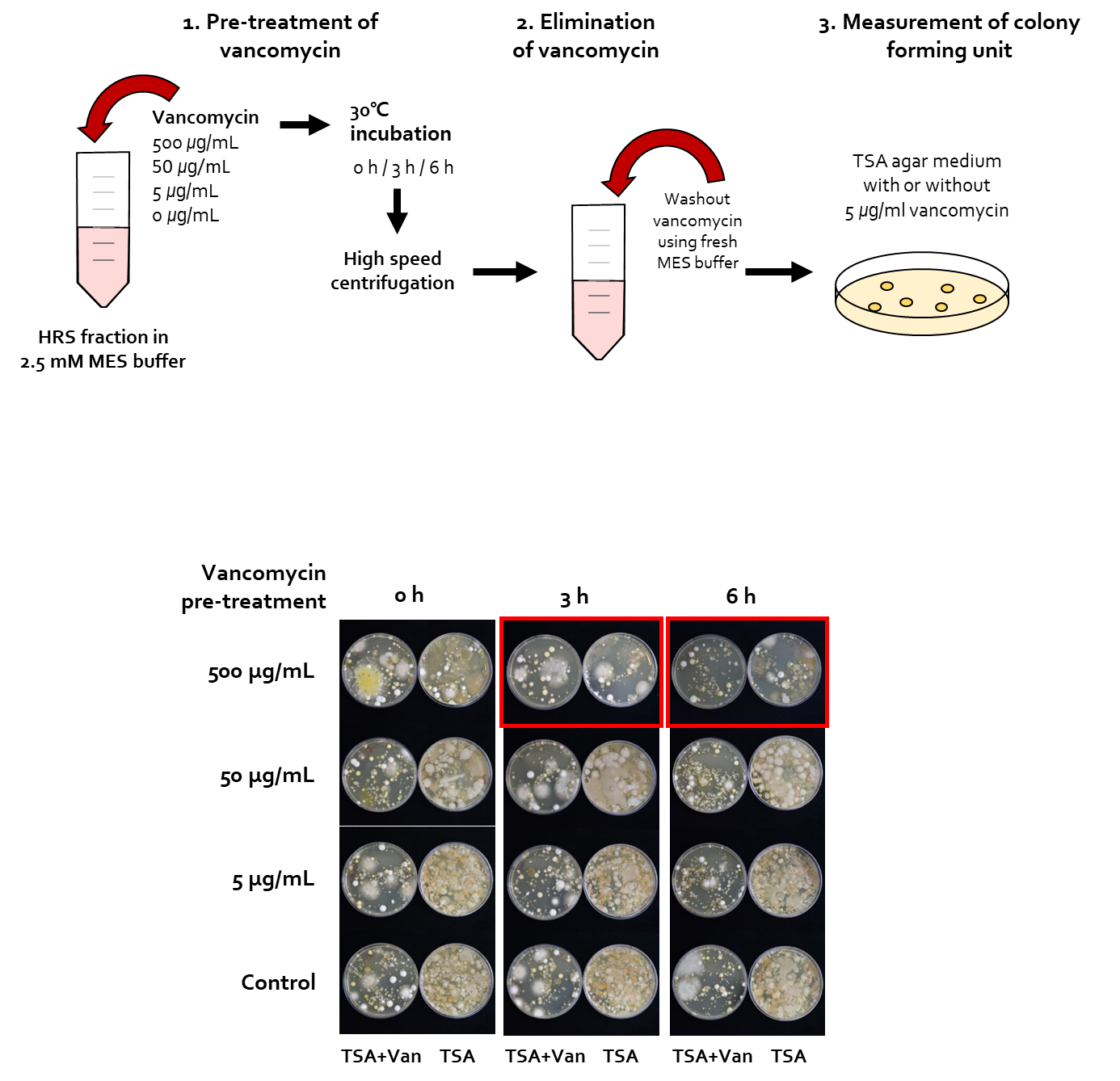
**

**b**

**Figure S2.** Optimization of vancomycin treatment using the HRS fraction. (a) Schematic representation of the vancomycin treatment and calculation of the colony-forming unit (CFU) values of bacterial isolates on TSA medium supplemented with or without 5 μg/mL vancomycin. (b) Comparison of bacterial CFU values of vancomycin-pretreated HRS fractions on TSA medium supplemented with or without 5 μg/mL vancomycin. Optimum vancomycin concentration and incubation time were 500 μg/mL and 3 h.

**
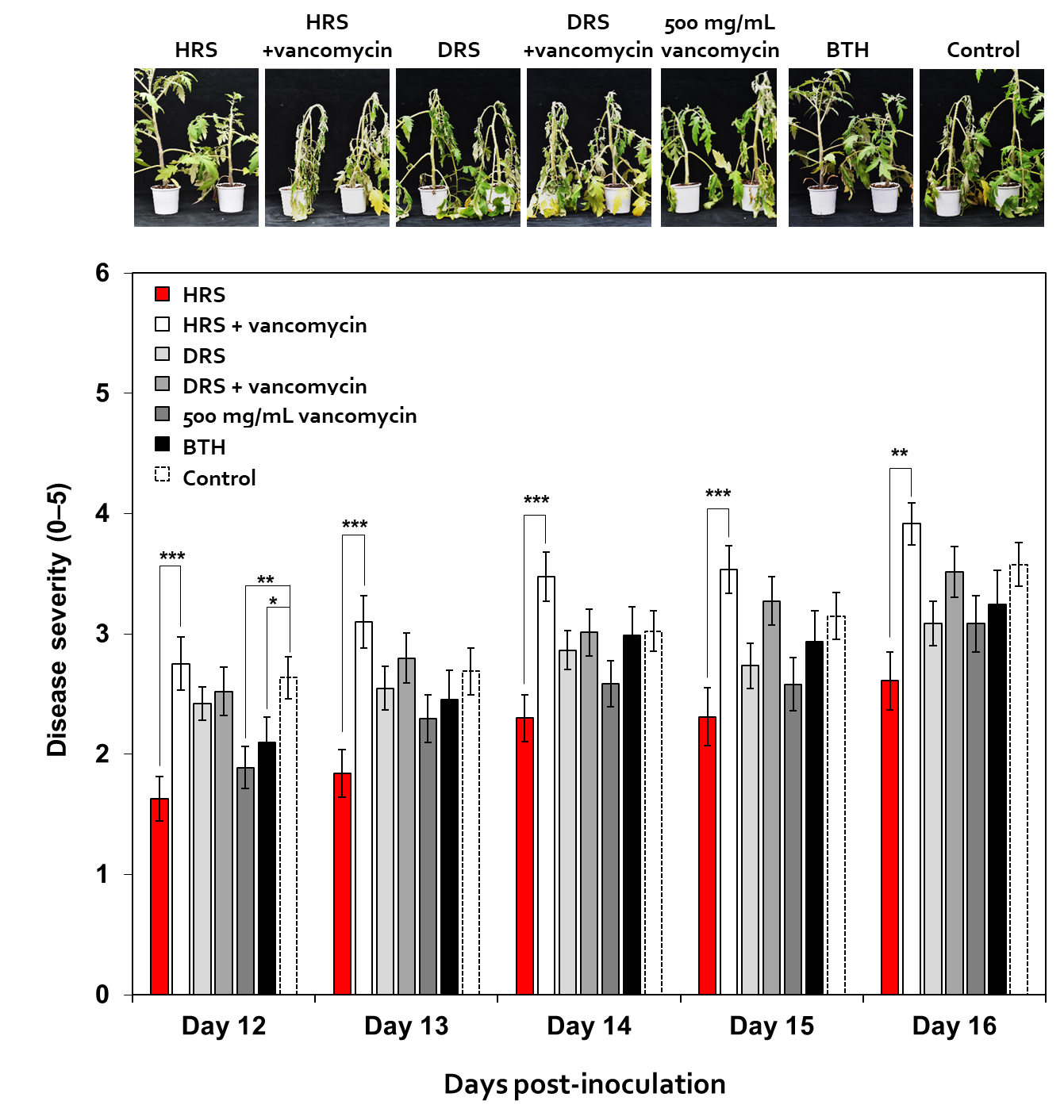
**

**Figure S3.** Disease severity in tomato plants treated with HRS and DRS fractions, with or without vancomycin pretreatment, at 12–16 days post-inoculation (dpi). HRS, HRS treatment; HRS + vancomycin, HRS fraction pretreated with vancomycin (500 µg/mL); DRS, DRS treatment; DRS + vancomycin, DRS fraction pretreated with vancomycin (500 µg/mL); 500 mg/mL vancomycin, root dipping treatment with 500 mg/mL vancomycin. Data represent mean ± SEM (*n* = 12 plants per treatment). Asterisks indicate significant differences (**P* < 0.05, ***P* < 0.01, ****P* < 0.001).

**
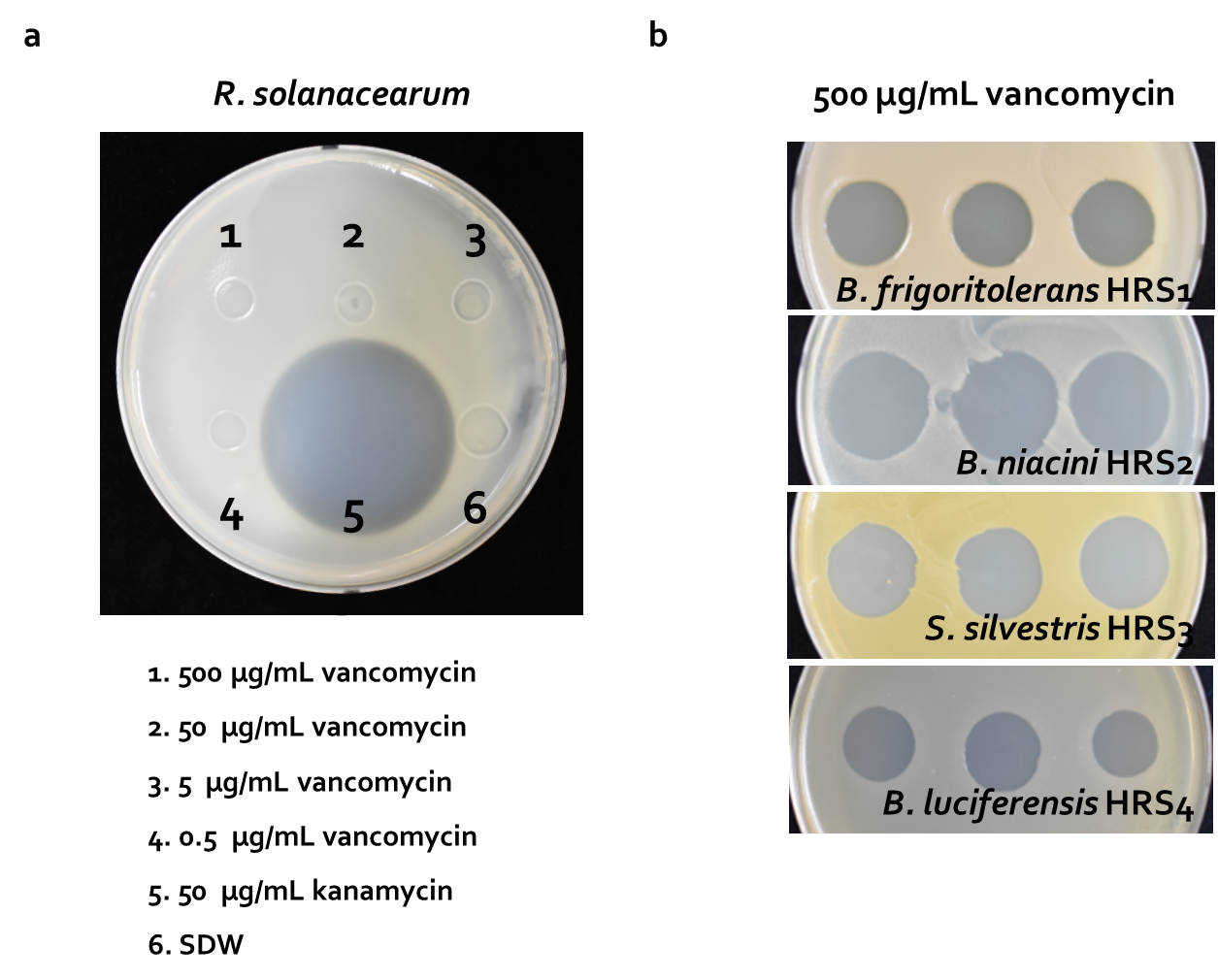
**

**Figure S4.** Effect of vancomycin on *Ralsonia solanacearum*, the causal organism of bacterial wilt, and four HRS-specific bacterial isolates. (a, b) Application of vancomycin (0.5, 5, 50, and 500 µg/mL) on TSA medium inoculated with *R. solanacearum* (a) or with each of the four HRS-specific bacterial strains (b). Kanamycin (50 µg/mL) was used as a positive control, and sterilized distilled water was used as a negative control.

**
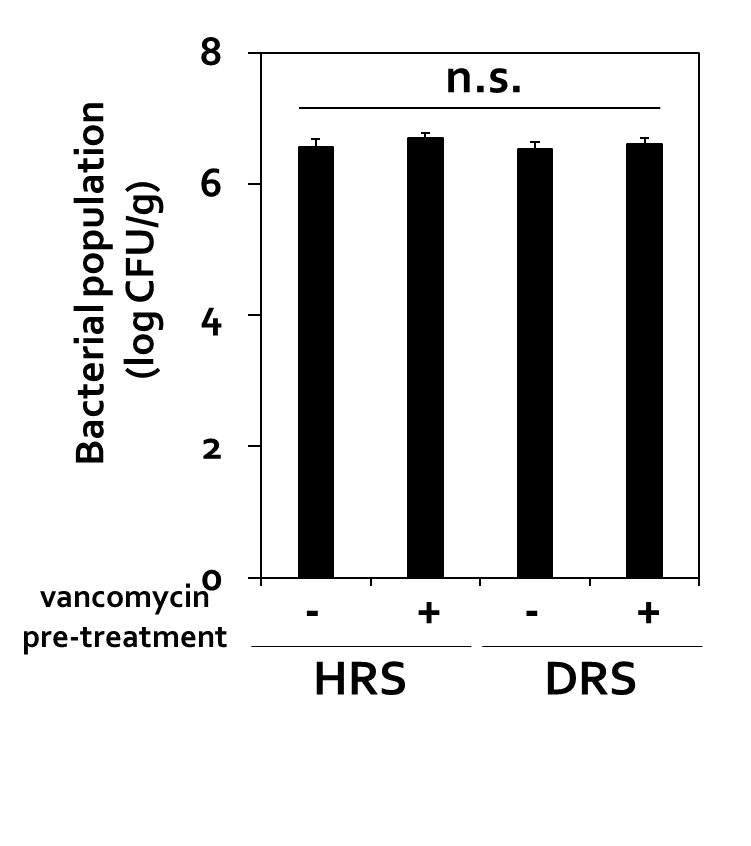
**

**Figure S5.** Quantification of the number of colony-forming units (CFU) of bacterial isolates in HRS and DRS fractions pretreated with or without 500 µg/mL vancomycin. “+” indicates vancomycin pretreated soil fractions, and “-” indicates vancomycin-free soil fractions.


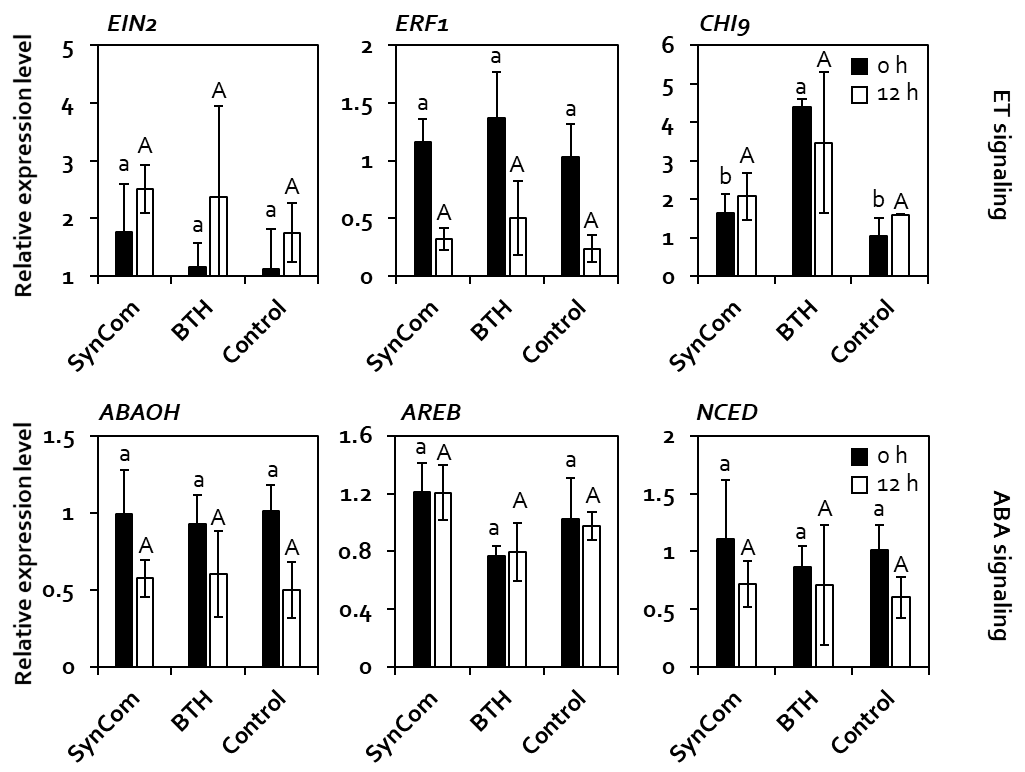


**Figure S6.** Relative expression levels of ethylene (ET) and abscisic acid (ABA) signaling marker genes in systemic leaves of tomato plants treated with a synthetic community (SymCom) of four HRS-specific strains at 0 and 12 h post-inoculation (hpi) with *Ralsonia solanacearum*. SynCom, a mixture of *Brevibacterium frigoritolerans* (HRS1), *Bacillus niacini* (HRS2), *Solibacillus silvestris* (HRS3), and *Bacillus luciferensis* (HRS4); BTH, 0.5 mM benzothiadiazole treatment; control, 2.5 mM MES buffer treatment. Data represent mean ± SEM. Different letters indicate significant differences among treatments (*P* < 0.05; least significant difference [LSD] test).
